# Cardiovascular and All-Cause Mortality Is Affected by Serum Magnesium and Diet Pattern in a Cohort of Dialysis Patients

**DOI:** 10.3390/jcm13144024

**Published:** 2024-07-10

**Authors:** Ioannis Petrakis, Dimitra Bacharaki, Periklis Kyriazis, Olga Balafa, Evangelia Dounousi, George Tsirpanlis, Marios Theodoridis, Ourania Tsotsorou, Anastasia Markaki, Anastasia Georgoulidou, George Triantafyllis, Ioannis Giannikouris, Apostolos Kokkalis, Aristeides Stavroulopoulos, Kostas Stylianou

**Affiliations:** 1 Nephrology Department, University General Hospital of Heraklion, 71500 Heraklion, Greece; petrakgia@gmail.com; 2 Nephrology Department, Attikon University Hospital, 12462 Athens, Greece; bacharaki@gmail.com (D.B.); raniadts@yahoo.gr (O.T.); 3 Division of Nephrology, Beth Israel Deaconess Medical Center, Harvard Medical School, Boston, MA 02215, USA; 4 Nephrology Department, University Hospital of Ioannina, 45500 Ioannina, Greece; olgabalafa@gmail.com (O.B.); edounous@uoi.gr (E.D.); 5 Nephrology Department, General Hospital of Athens “G. Gennimatas”, 11527 Athens, Greece; tsirpanlis@yahoo.gr; 6 Department of Nephrology, Democritus University of Thrace, 68150 Alexandroupolis, Greece; cymarth@otenet.gr; 7 Department of Nutrition and Dietetics, Hellenic Mediterranean University, 71410 Heraklion, Greece; markakin@hotmail.com; 8 Nephrology Department, General Hospital of Komotini, 69133 Komotini, Greece; ana.georgoulidou@yahoo.gr; 9 Department of Nephrology, Hemodialysis Unit, Mediterraneo Hospital, 16675 Glyfada, Greece; triantafyllisg@gmail.com (G.T.); ioannis.e.giannikouris@gmail.com (I.G.); 10 Ionio Salaminas Hemodialysis Center, 18900 Salamina, Greece; ackokkalis@hotmail.com; 11 Nephrology Department, General Clinic of Kalithea, IASIO Hospital, 17675 Athens, Greece; stavroulopoulos@yahoo.co.uk; 12 NEPHROEXPERT—Athens Kidney Institute, 17675 Athens, Greece

**Keywords:** Mediterranean diet, magnesium, hemodialysis, peritoneal dialysis, mortality, cardiovascular

## Abstract

**Background:** Hypomagnesaemia is associated with an increased overall mortality in patients with chronic kidney disease on dialysis (CKD-5D). Mediterranean-style diet (MD), having a high magnesium content, can serve as a form of dietary magnesium supplementation. We examined whether there is a potential link between increased Mediterranean Diet score (MDS) and elevated serum magnesium (sMg) to assess its impact on reducing mortality risk in CKD-5D patients. **Methods:** In this multi-center prospective observational study, 117 CKD-5D patients (66 on hemodialysis and 51 on peritoneal dialysis) with a mean age of 62 ± 15 years were studied for a median follow-up period of 68 months. After baseline assessment, including measurement of sMg and MDS, all patients were followed up for cardiovascular (CV) and all-cause mortality. **Results:** Forty deaths occurred, 58% of which were cardiovascular. Patients who were above the median value of sMg (2.2 mg/dL) had a 66% reduction in CV (crude HR, 0.34; 95% CI, 0.11–0.70), and 49% reduction in all-cause (crude HR, 0.51; 95% CI, 0.27–0.96) mortality, even after adjustment for age, malnutrition inflammation score, left ventricular mass index, peripheral vascular disease and diabetes. Similar results were obtained when sMg was analyzed as a continuous variable. sMg was associated directly with MDS (r = 0.230; *p* = 0.012). **Conclusions:** Higher sMg levels are strongly and independently associated with reduced CV and all-cause mortality in CKD-5D patients. A strong correlation exists between MDS and sMg. Elevated sMg levels, achieved through MD adherence, can significantly reduce CV mortality, implicating MD as a mediator of the association between sMg and CV mortality.

## 1. Introduction

Patients with end-stage kidney disease (ESKD) face an unprecedented burden of cardiovascular diseases (CVDs) [1]. The prevailing association between CVDs and ESKD leads to the overall survival of hemodialysis (HD) patients or peritoneal dialysis (PD) patients of approximately 50% in 5 years [2,3]. Traditional risk factors of CVD such as diabetes mellitus (DM), arterial hypertension (AH) and dyslipidemia are highly prevalent in ESKD patients [4]. Uremia-related risk factors, such as oxidative stress, inflammation, endothelial dysfunction and chronic kidney disease–mineral bone disease (CKD–MBD), arise as important mediators of CVD-related or overall mortality [5,6].

Abundant experimental evidence highlights magnesium’s involvement in numerous biological processes within the vascular system. A magnesium-rich environment induces monocyte immunomodulation towards an anti-inflammatory activation pattern [7]. Extracellular matrix calcification is attenuated through magnesium autophagy induction [8]. Collectively, mild hypermagnesemia reduces soft tissue and vascular calcifications [9]. A magnesium-rich diet was associated with lower coronary artery calcification in asymptomatic individuals participating in the Framingham Heart Study [10]. Magnesium supplementation could promote a more favorable CKD–MBD phenotype acting both on the Ca^+2^–Parathormone axis and on carotid intima media thickness among CKD-5D patients [11].

Low serum magnesium (sMg) has been associated with increased CVD- and all-cause mortality both in HD and PD patients [9,12,13]. There are few recommendations on therapeutic interventions for restoring magnesium metabolism and related outcomes in CKD-5D patients [14]. Dietary magnesium intake has been pinpointed as a potential inhibitor of CKD progression [15,16,17].

Mediterranean Diet and DASH diet have been associated with lower mortality in the CKD-5D population [18]. Magnesium constitutes a key ingredient in both the Mediterranean Diet as well as in the DASH diet [19,20]. However, in MAGiCAL-CKD, a randomized trial examining the effect of oral magnesium supplementation on vascular calcification, there was no difference in the mortality of CKD patients [21].

The current study examines the impact of sMg acquired through a Mediterranean-style diet (MD) on CVD- and all-cause mortality in a cohort of CKD-5D patients.

## 2. Material and Methods

### 2.1. Study Population

The study was conducted in nine peritoneal/hemodialysis units across Greece. Participants were included if they had been on dialysis for at least three months and were at least 18 years of age. Those who had undergone major surgery recently, had a malignant disease, were experiencing concurrent inflammatory illness, had a life expectancy below 12 months, had cognitive impairment or were unwilling to participate were excluded. A total of 117 eligible CKD-5D patients (66 HD and 51 PD) were recruited between July 2016 and December 2017. The treatment regimen for HD patients involved three sessions per week, each lasting a minimum of four hours. The therapy utilized a bicarbonate dialysis solution with the goal of achieving a minimum target KT/V of 1.2. as recommended [22]. The PD group consisted of individuals undergoing continuous ambulatory PD (4–5 exchanges per day) or individuals on automated peritoneal dialysis as described [23]. All HD patients were treated with a dialysate Mg (dMg) concentration of 0.5 mmol/L, whereas 22 and 29 PD patients were treated with a dMg concentration of 0.25 and 0.50 mmol/L, respectively. The dMg prescription remained constant during the follow-up period. The study was performed in strict accordance with the ethical guidelines of the Helsinki Declaration and was approved by the Ethical Scientific Committee of the participating centers. All study participants provided written informed consent.

### 2.2. Clinical Data

The assessment and collection of clinical data have been previously described [24] and are presented as follows. A comprehensive review of each patient’s medical chart was conducted with the objective of extracting data on a range of cardiovascular conditions, including vascular disease (PVD), coronary artery disease (CAD), stroke, diabetes and arterial hypertension. Additionally, the review included an analysis of the prescribed antihypertensive medications, lipid-lowering agents (statins) and calcimimetics. Baseline PVD was defined as the development of symptoms (intermittent claudication), therapeutic interventions (revascularization and amputation), and artery stenosis > 60% in imaging studies. Coronary artery disease (CAD) was defined as a medical history of myocardial infarction, angina pectoris, percutaneous coronary intervention and coronary artery bypass surgery. Arterial hypertension was identified either by measuring systolic blood pressure exceeding 140 mmHg or by the use of antihypertensive medication within six months of enrolment. Echocardiographic studies were performed before the midweek dialysis session for hemodialysis (HD) patients and on the day of the scheduled visit for peritoneal dialysis (PD) patients. Left ventricular hypertrophy (LVH), as indicated by an LV mass index (LVMI) exceeding 95 g/m^2^ in female patients and 115 g/m^2^ in male patients, was identified as an echocardiographic criterion. The LV mass was quantified in accordance with the Devereux formula [25]. Baseline clinical information was also collected for each patient, including age, dialysis vintage and body mass index (BMI).

### 2.3. Mediterranean Diet Score

Adherence to the Mediterranean diet (MD) was assessed using the Med diet score (MDS; Table 1), as described previously by Panagiotakos et al. [26] and Bacharaki et al. [24].

In summary, a registered dietitian was tasked with questioning patients about their consumption of a range of foods and beverages over the preceding week. These included non-refined cereals, fruits, vegetables, legumes, potatoes, fish, red meat and meat products, poultry, full-fat dairy products, olive oil and alcohol. The scoring system was designed to reflect the typical dietary patterns observed in the Mediterranean region. For foods presumed to align with a Mediterranean dietary pattern, including non-refined cereals, fruits, vegetables, legumes, olive oil, fish and potatoes, higher scores indicate increased consumption in alignment with this dietary pattern. Conversely, for foods presumed to deviate from this typical pattern, including red meat and products, poultry and full-fat dairy products, lower scores indicate greater adherence to these patterns. The total score is assigned a value between 0 and 55, with the highest score reflecting the highest level of adherence to the Mediterranean diet. To further investigate the relationship between individual food categories and important cardiovascular disease (CVD) risk factors, a separate analysis was conducted involving three groups: Group A is defined as the “avoid foods” group, which comprises the three ingredients that were attributed a negative value by incremental consumption (red meat and products, poultry and full-fat dairy products). Group B is defined as the “recommended foods” group, which encompasses the seven ingredients that were assigned a positive value by incremental consumption (non-refined grains, potatoes, fruits, vegetables, legumes, fish and olive oil). Group C is the “fruit, vegetables, legumes-FVL” group.

### 2.4. Malnutrition–Inflammation Score

Malnutrition–inflammation score (MIS), as described by Kalantar-Zadeh et al. [27] and Bacharaki et al. [24], was calculated for all patients. The Malnutrition Screening Tool (MIS) comprises four domains, encompassing the assessment of the patient’s medical history, physical examination, body mass index (BMI), and laboratory parameters, along with 10 individual components. A total score is calculated from all MIS components, with a possible range of 0–30. Higher scores indicate an increased risk of malnutrition and inflammation.

### 2.5. Laboratory Measurements

For laboratory testing, we employed the same methodology described in our earlier work [24]. In particular, blood samples were collected prior to the mid-week dialysis session for patients undergoing hemodialysis (HD) and on the morning of the scheduled visit for patients undergoing peritoneal dialysis (PD). The following parameters were measured on a routine basis at least once a month: hemoglobin (Hb), serum albumin (sAlb), creatinine, total cholesterol, high-density lipoprotein (HDL) and low-density lipoprotein (LDL) cholesterol, triglycerides, serum calcium (sCa), serum magnesium (sMg), serum phosphorus (sP) and C-reactive protein (CRP). Serum parathormone (PTH) was measured at least quarterly. All measurements were conducted using standard laboratory techniques. The average of the laboratory data from the previous three months was employed for the analysis.

### 2.6. Follow-Up

Follow-up data were retrieved from clinical records and/or death certificates by the attending nephrologists. Follow-up began on the date of enrolment and finished upon the death (CVD-associated or all-cause) of the patient or on 30 June 2023, whichever came first. CVD-related deaths included deaths as a result of coronary heart disease, sudden death, stroke, or complicated peripheral vascular disease (septic shock from critical limb ischemia and/or gangrene and fatal amputation). During the follow-up period, eleven patients who started on PD had switched to HD and nine patients underwent a kidney transplantation. No patient was lost to follow-up.

### 2.7. Statistical Analysis

All statistical analyses were conducted using the SPSS/PC 22 statistical package (Chicago, IL, USA). Normally distributed variables were expressed as the mean ± standard deviation, while non-normally distributed variables were expressed as the median (interquartile range). sMg was examined both as a continuous and as a dichotomous variable; in the latter case, patients were classified into two groups: those who were below the median value of sMg (<2.2 mg/dL) (Low Mg group) and those who were above the median value of sMg ≥ 2.2 mg/dL (High Mg group). Differences in baseline characteristics between the groups were tested using the χ^2^ test and the Kruskal–Wallis test as appropriate. Univariate and multivariate regression analyses were used to determine associations between variables.

Survival analysis was performed using Kaplan–Meier survival curves. Cox proportional hazards models were used to evaluate the relationship between sMg and mortality (CVD and all-cause), initially without adjustment and subsequently adjusting for variables related to sMg at baseline (dMg, MDS or ‘recommended foods’ (one of the categories of MDS) and for traditional risk factors univariately associated with CVD and all-cause mortality at the *p* < 0.05 level. Statistical significance was set at the level of *p* < 0.05 (two-sided).

## 3. Results

### 3.1. Study Population

The study cohort comprised 117 patients with a mean age of 62 ± 15 years (range 20–83), as shown in Table 2. A total of 66 patients (46 males and 20 females with a mean age of 62 ± 16 years) were undergoing hemodialysis (HD) treatment, while 51 patients (25 males and 26 females with a mean age of 63 ± 14 years) were treated with peritoneal dialysis (PD). The prevalences of diabetes, PVD, CAD and stroke were 27.4%, 38.5%, 28.2% and 16.2%, respectively. Of the 117 patients with hypertension, 90 (76.9%) were receiving antihypertensive medication. The majority of these patients (*n* = 81) were taking beta-blockers (*n* = 78), calcium channel blockers (*n* = 47), angiotensin-converting enzyme inhibitors/angiotensin receptor blockers (*n* = 49) and diuretics (*n* = 30). A total of 59 and 38 patients were statin and calcimimetics users, respectively. Dialysis vintage was shorter in PD than in HD patients (44 ± 37 vs. 78 ± 50 months; *p* < 0.05).

### 3.2. Determinants of Serum Magnesium (sMg) Levels

**Figure 1:**
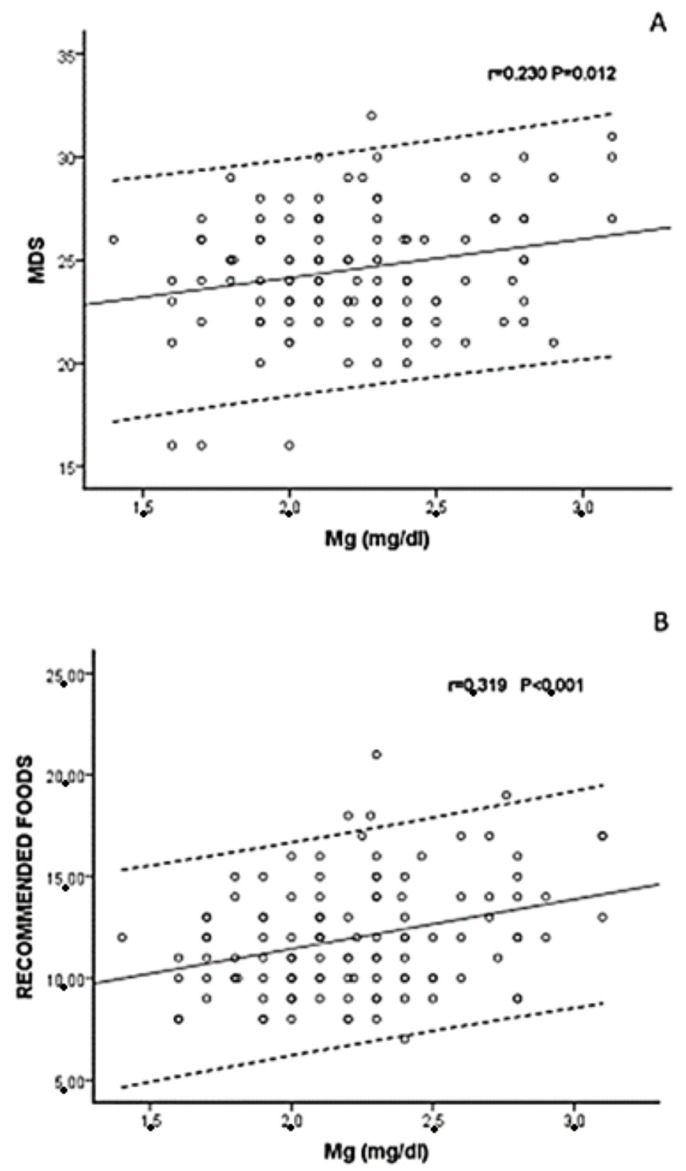
The correlations between serum Mg (mg/dL) and (**A**) Mediterranean diet score (MDS) and (**B**) consumption of recommended foods. Overlapping circles indicate similar values out of 117 patients. Dotted lines indicate 95% C.I. (confidence interval) of the correlation (continuous black line).

As shown in Table 2, sMg was positively associated with dialysis mode, dMg, serum albumin, HDL, use of ACEIs/ARBs, MDS and Med diet food categories: recommended food and ‘FVL’. The associations between sMg and MDS (r = 0.230) and consumption of recommended foods (r = 0.319) are shown in Figure 1.

The results of a multivariate regression analysis (Table 3), where variables significant in univariate analysis were included, showed that only dMg, recommended foods and dialysis mode were independently associated with sMg. Given that the correlation (r) between ‘recommended foods’ and sMg was higher than the corresponding correlation between MDS and sMg, we opted to include only recommended foods in our regression model. This model explained 29% of the variability in sMg levels.

To further explore the correlation between sMg and the Mediterranean diet in patients with CKD stage 5, we analyzed the correlations between sMg and MDS, along with its categories, separately, in our 61 HD and 51 PD patients. In the HD patient group, sMg correlated positively with MDS (r = 0.247; *p* = 0.045), ‘recommended foods’ (r = 0.362; *p* = 0.002) and ‘FVL’ (r = 0.283; *p* = 0.021), and negatively with ‘avoid foods’ (r = −0.129; *p* = 0.302). Among the PD patients, sMg correlated positively with MDS (r = 0.177; *p* = 0.214), ‘recommendation foods’ (r = 0.33; *p* = 0.017) and ‘FVL’ (r = 0.287; *p* = 0.041), and negatively with ‘avoid foods’ (r = −0.054; *p* = 0.705).

### 3.3. Associations between Baseline Clinical and Biochemical Characteristics and MDS and Mediterranean Diet Food Categories

Since the role of MDS was of prime importance in the association between sMg and mortality in our study, we next explored the relationship between baseline clinical and biochemical characteristics and MDS and Mediterranean diet food categories. As shown in Table 4, adherence to the MDS was associated with a lower prevalence of LVH and PVD, lower LVMI, SBP, DBP and serum Ca, and higher HDL. Consumption of recommended foods was associated with a lower prevalence of LVH and PVD, and lower DBP and LDL. A subset of recommended foods included in group C (FVL) was associated with a lower prevalence of diabetes, stroke and LVH. Lower and higher use of beta-blockers and calcimimetics, respectively, were associated with adherence to MDS, consumption of recommended foods and FVL. Consumption of ‘avoid foods’ was associated with a higher prevalence of CAD.

### 3.4. Serum Magnesium (sMg) Levels and Mortality

**Figure 2:**
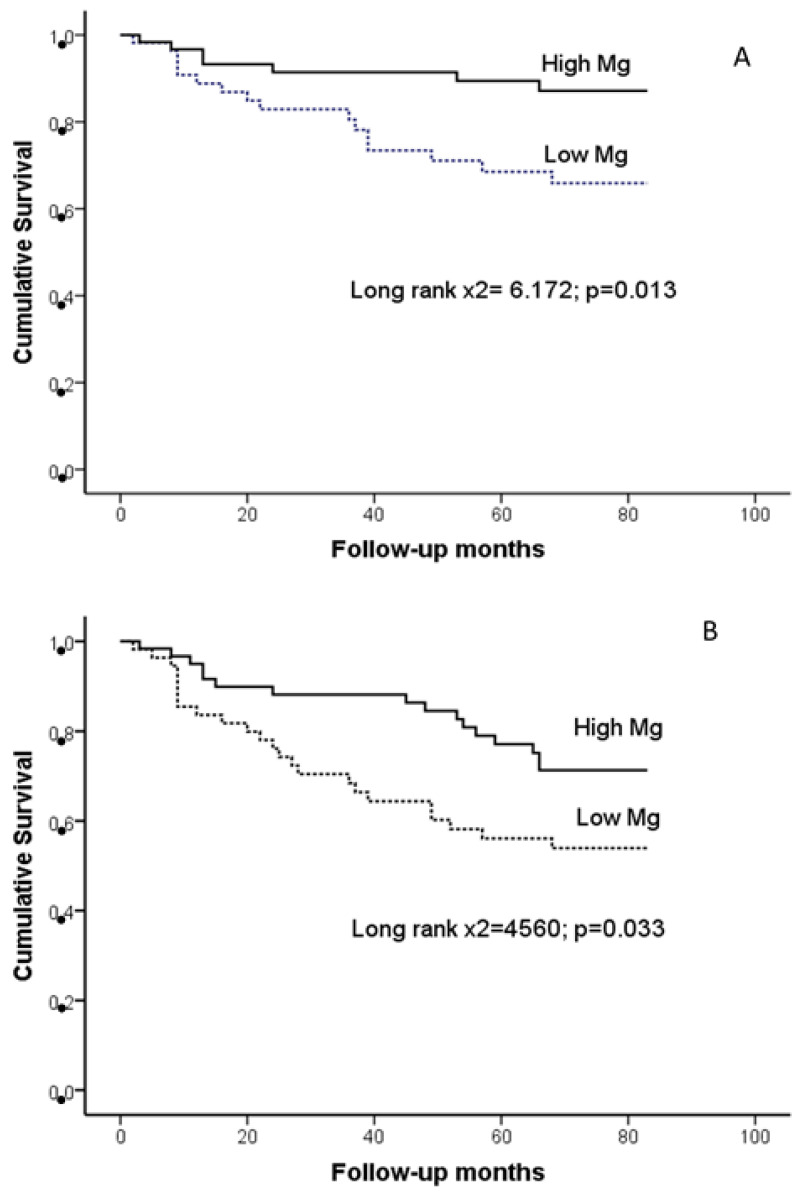
Kaplan–Meier survival analysis of (**A**) cardiovascular and (**B**) all-cause mortality in patients with serum magnesium (sMg) levels above the median (High Mg) compared with those with levels below the median (low Mg).

Over a median follow-up period of 68 months, 40 deaths occurred, 23 (58%) of which were cardiovascular. Patients who died from CVDs had lower sMg compared with surviving patients (2.03 ± 0.27 vs. 2.26 ± 0.37 mg/dL; *p* = 0.007). Kaplan–Meier survival analysis (Figure 2) using the median of sMg levels (low vs. high Mg group) revealed that patients in the low Mg group had a reduced survival rate in comparison to those in the high Mg group, impacting both CVD (*p* = 0.013) and all-cause mortality (*p* = 0.033) outcomes. The association between sMg and CVD or all-cause mortality was studied using univariate and multivariate Cox analysis (Table 5).

In unadjusted Cox regression analysis, every 1 mg/dL of increase in sMg decreased CVD and all-cause mortality risk by 83% (crude HR, 0.17; 95% CI, 0.05–0.62) and 73% (crude HR, 0.27; 95% CI, 0.11–0.70), respectively. This decreased risk persisted even after adjustment for creatinine, albumin, MIS, LVMI, PVD, diabetes and age. However, adjustment for MDS attenuated the CVD but not all-cause mortality association with sMg levels. All the above factors that were entered as covariates into the multivariate models were univariately significant (all *p* < 0.05) predictors of both CVD and all-cause mortality.

Next, sMg was examined, using Cox regression, as a dichotomous variable comparing the low Mg group (below the median of sMg) with the high Mg group (above the median of Mg) (Table 6). In unadjusted Cox regression analysis, patients in the high Mg group (sMg ≥ 2.2 mg/dL) had a 66% reduction in CVD (crude HR, 0.34; 95% CI, 0.14–0.83) and 49% reduction in all-cause (crude HR, 0.51; 95% CI, 0.27–0.96) mortality compared to the low Mg group. This association persisted even after adjustment for all the variables mentioned above, but further adjustment for MDS resulted in a considerable loss of the significance of the association of the sMg groups with CVD, but not with all-cause mortality.

Finally, a subgroup analysis was conducted to examine outcome differences between HD (Table 7) and PD (Table 8) patients. In the unadjusted Cox regression analysis of HD patients, each 1 mg/dL increase in sMg reduced the risk of CVD and all-cause mortality by 88% (crude HR, 0.12; 95% CI, 0.02–0.85) and 81% (crude HR, 0.19; 95% CI, 0.04–0.86), respectively. For PD patients, each 1 mg/dL increase in sMg was associated with an 84% reduction in CVD mortality (crude HR, 0.16; 95% CI, 0.03–0.98) and a 72% reduction in all-cause mortality (crude HR, 0.28; 95% CI, 0.08–0.96). These reductions in risk for both HD and PD patients persisted even after adjusting for factors associated univariately with CVD and all-cause mortality. The association between sMg and mortality (both CVD and all-cause) in both HD and PD groups disappeared after adjusting for “recommended foods”, a category within the Mediterranean diet score (MDS) that has the strongest correlation with sMg among all MDS categories and the overall MDS itself (Table 2). Significantly, elevated calcium levels in the HD group and reduced potassium levels in the PD group were associated with a higher risk of CVD and all-cause mortality. Both serum calcium and potassium were included as covariates in the respective Cox analyses. Comparing outcomes between the HD and PD groups, unadjusted Cox regression analysis showed a slightly higher reduction in both CVD and all-cause mortality in the HD group compared to the PD group.

## 4. Discussion

The present study showed that patients in the high Mg group (sMg ≥ 2.2 mg/dL) displayed a significantly lower risk of CV and all-cause mortality as compared to those in the low Mg group (sMg < 2.2). The same results were obtained when sMg was analyzed as a continuous variable, where for each 1 mg/dL increment in sMg there were 83% and 73% reductions in CVD and all-cause mortality, respectively. In both analyses, this sMg–mortality (CVD and all-cause) relationship remained significant even after adjustment for a number of significant predictors of both CVD and all-cause deaths. However, after adjusting for MDS, sMg lost its prognostic value for CVD deaths, but not for all-cause deaths, thus implicating MDS as a potential mediator of the observed association between sMg and CVD mortality. Given that MDS was directly associated with sMg levels, the Mediterranean eating pattern could serve as an effective tool for magnesium repletion in CKD-5D patients, thus resulting in substantial improvements in CVD survival.

CKD-5D patients suffer from intracellular magnesium depletion [28], and hypomagnesemia remains an independent risk factor for CVD and overall mortality in many CKD cohorts [9,12,29,30]. In CKD-5D, hypomagnesemia contributes to CVD and overall mortality irrespective of the dialysis modality [13,30,31]. Lower serum magnesium has been associated with poorer nutritional status in multiple cohorts of peritoneal dialysis patients [13,31]. In a cohort study with a sample size of 1100 adults, the CKD-5D risk was reduced when better compliance with the DASH diet pattern was present [32]. Magnesium intake was a strong mediator of the aforementioned relationship [15]. Magnesium supplementation has been proposed as a means of hypomagnesemia correction [30]. Oral magnesium supplementation is an efficient means of magnesium repletion [21]. Mild hypermagnesemia may have a protective effect in reducing overall mortality [33]. However, in the CANVAS trial, magnesium levels did not affect cardiovascular outcomes [34]. The CANVAS study included patients suffering from CKD stage 3 or milder. CKD 4 and 5 patients were not included in the study. All patients had a prior history of established diabetes mellitus type 2 and CVDs. The authors comment that the mean difference in serum magnesium between patients receiving canagliflozin and those receiving placebo might not have been high enough to show biological significance above that offered by canagliflozin [34]. Analyzing further the role of serum magnesium and dietary magnesium supplementation, a meta-analysis performed by Del Gobbo et. al. [35] demonstrated that circulating magnesium was associated with a 30% lower risk of CVD (per 0.2 mmol/L sMg increment) and dietary magnesium supplementation reduced the risk of ischemic heart disease by 22%. Of note, the aforementioned study did not include prevalent CKD patients. The present study provides evidence that the relationship between serum magnesium (sMg) and mortality (both CVD and all-cause) in both hemodialysis (HD) and peritoneal dialysis (PD) groups is not statistically significant after adjusting for “recommended foods”, a category within the MDS that has the strongest correlation with sMg among all MDS categories and the overall MDS itself. This finding further elucidates the underlying mechanism of the sMg–mortality relationship. In this context, our results suggest that future well-designed studies could investigate whether sMg might serve as a biomarker of adherence to the Mediterranean diet.

Considering that intracellular magnesium stores appear to be independent of serum magnesium concentrations [36], the mechanisms contributing to the observed benefits of dietary magnesium supplementation in CKD-5D patients may be associated with restoring both extra- and intra-cellular magnesium concentrations with MD. As of January 2024, around 25 clinical studies registered at clinicaltrials.gov have examined the effect of magnesium supplementation in conjunction with clinical outcomes in CKD patients. The vast majority of these studies have no published results.

Increased parathormone and elevated serum phosphate are implicated in overall and cardiovascular mortality [37,38]. Surprisingly, in this study, phosphate and parathormone did not show any association with cardiovascular and overall mortality. Similarly, IMPROVE-CKD, a multicentric, randomized control trial exploring the effects of phosphate-lowering in CKD patients failed to show benefits in terms of CVD outcomes [39]. In their meta-analysis of 309 patients, Guo et al. found that magnesium supplementation could reduce PTH and carotid intima-media thickness in hemodialysis patients [11]. In the present cohort, serum magnesium and magnesium-rich diet outperformed serum phosphate and parathormone in terms of overall and CVD-associated mortality.

Our study has limitations as an observational study without a randomization arm. The focus on the Mediterranean eating pattern as a mediator of the association between serum magnesium (sMg) and mortality risk is specific to an ethnic group, impacting its generalizability. Furthermore, the present study did not examine the impact that physical activity levels, socioeconomic status, medication adherence and their associations with sMg or mortality may have. Despite these limitations, the study robustly established a connection between sMg levels and cardiovascular disease (CVD) mortality in 117 CKD-5D patients. Furthermore, it confirmed that the dietary supplementation of magnesium is the underlying mechanism driving this association. While relying on a single baseline sMg measurement raises concerns, the stability of dialysate magnesium concentrations and dietary patterns throughout the observational period underscores their consistent influence on sMg levels. Future research with larger cohorts including a magnesium supplementation cohort driven by a Mediterranean-style diet compared with a placebo will have the potential to shed light on the causal relationship between diet, magnesium, and mortality.

In conclusion, adopting a high dietary magnesium intake may act as a strategy to facilitate magnesium repletion, potentially improving mortality outcomes in CKD-5D patients.

## Figures and Tables

**Table 1:** The Mediterranean diet score.

How Often do You Consume (Servings/Week or Otherwise Stated)	Frequency of Consumption					
**Non-refined cereals (whole grain bread, pasta, rice, etc)**	**Never**	**1–6**	**7–12**	**13–18**	**19–31**	**>32**
	0	1	2	3	4	5
Potatoes	Never	1–6	5–8	9–12	13–18	>18
	0	1	2	3	4	5
Fruits	Never	1–4	5–8	9–15	16–21	>22
	0	1	2	3	4	5
Vegetables	Never	1–6	7–12	13–20	21–32	>33
	0	1	2	3	4	5
Legumes	Never	<1	1–2	3–4	5–6	>6
	0	1	2	3	4	5
Fish	Never	<1	1–2	3–4	5–6	>6
	0	1	2	3	4	5
Red meat and products	≤1	2–3	4–5	6–7	8–10	>10
	5	4	3	2	1	0
Poultry	≤3	4–5	5–6	7–8	9–10	>10
	5	4	3	2	1	0
Full-fat dairy products (cheese, yogurt, milk)	≤10	11–15	16–20	21–28	29–30	>30
	5	4	3	2	1	0
Use of olive oil for cooking (times/week)	Never	Rare	<1	1–3	3–5	Daily
	0	1	2	3	4	5
Alcoholic beverages (100 mL = 12 g ethanol)	<300	300	400	500	600	>700 or 0
	5	4	3	2	1	0

**Table 2:** Characteristics of the patients classified into low (<2.2 mg/dL) and high (≥2.2 mg/dL) serum magnesium (sMg) level groups.

**Characteristics**	**All Patients** **N = 117**	**Low sMg** **N = 56**	**High sMg** **N = 61**	***p* ^a^**	**r †**	***p* ^b^**
**Epidemiological and Clinical**						
Age (year)	62 ± 15	64 ± 15	61 ± 15	0.257	−0.178	0.055
Sex (male = 1; females = 2 (*n*)	71/46	23,620	43/33	0.445	0.099	0.288
Dialysis mode (HD/PD) (*n*)	66/51	35/21	35/26	0.203	0.187	**0.043**
dMg (0.25/0.50 mmol/L (*n*)	22/95	16/40	6/55	**0.010**	0.245	**0.008**
Dialysis vintage (months)	37 (18–63)	41 (22–67)	32(16–58)	0.203	−0.076	0.413
Diabetes mellitus (%)	27.4	30.4	24.6	0.485	−0.114	0.222
Hypertension (%)	76.9	76.7	77	0.973	−0.054	0.565
Cardiac artery disease (%)	28.2	39.9	22	0.187	−0.186	**0.044**
Peripheral artery disease (%)	38.5	46.4	31.1	0.090	−0.281	**0.002**
Stroke (%)	16.2	19.6	13.1	0.339	−0.186	**0.044**
Left ventricular hypertrophy (%)	65.8	80.4	55.2	**0.001**	−0.323	**0.000**
LVMI (g/m^2^)	115 ± 29	120 ± 27	110 ± 29	0.053	−0.190	**0.040**
Body mass index (Kg/m^2^)	26.3 ± 4.8	28.9 ± 5.1	25.7 ± 4.5	0.207	−0.168	0.070
Systolic blood pressure (mmHg)	133 ± 19	132 ± 18	135 ± 20	0.344	0.037	0.690
Diastolic blood pressure (mmHg)	76 ± 14	75 ± 13	78 ± 15	0.229	0.066	0.481
Pulse pressure (mmHg)	57 ± 15	57 ± 14	57 ± 15	0.932	−0.014	0.879
**Drugs**	**All patients**	**Low sMg**	**High sMg**	***p* ^a^**	**r †**	***p* ^b^**
ACEIs/ARBs (%)	41.9	39.3	44.3	0.586	0.182	**0.050**
β-Βlockers (%)	67.2	67.9	66.7	0.891	−0.121	0.194
CCB (%)	40.5	35.7	45	0.309	0.100	0.287
Diuretics (%)	25.6	25	26.2	0.879	−0.005	0.958
Calcimimetics (%)	32.5	30.4	34.4	0.639	0.123	0.188
Statins (%)	50.4	48.2	52.5	0.646	0.051	0.583
**Nutritional and biochemical**	**All patients**	**Low sMg**	**High sMg**	***p* ^a^**	**r †**	***p* ^b^**
Albumin (g/dL)	3.62 ± 0.49	3.56 ± 0.52	3.87 ± 0.46	0.177	0.220	**0.017**
Creatinine (mg/dL)	8.7 ± 3.0	8.6 ± 3.0	8.9 ± 2.9	0.609	0.106	0.259
Hemoglobin (g/dL)	12.3 ± 14	12.6 ± 4.5	12.0 ± 3.5	0.387	0.101	0.509
Total Cholesterol (mg/dL)	167 ± 32	154 ± 32	160 ± 31	0.259	0.109	0.242
HDL Cholesterol (mg/dL)	43 ± 11	40 ± 12	46 ± 10	**0.011**	0.251	**0.006**
LDL Cholesterol (mg/dL)	83 ± 25	80 ± 23	86 ± 27	0.162	0.154	0.097
Triglycerides (mg/dL)	140 (101–184)	144(102–212)	130 (99–174)	0.116	−0.139	0.135
Calcium (mg/dL)	9.2 ± 0.6	9.3 ± 0.6	9.1 ± 0.6	0.294	−0.057	0.544
Magnesium (mg/dL)	2.2 ± 04	1.9 ± 0.2	2.5 ± 0.3	**0.000**	-	-
Potassium (mmol/L)	4.7 ± 0.7	4.6 ± 0.7	4.7 ± 0.6	0.700	0.014	0.789
Phosphorus (mg/dL)	4.9 ± 1.3	4.9 ± 1.3	5 ± 1.3	0.671	0.057	0.542
Parathormone (pg/mL)	262 (151–402)	257 (145–405)	268 (155–395)	0.357	0.008	0.934
MIS	4 (3–6)	4 (3–6)	4 (3–6)	0.360	−0.069	0.461
CRP	0.79 (0.33–3)	0.86 (0.33–2.80)	0.70 (0.32–3.14)	0.160	−0.118	0.209
MDS	25 ± 3	24 ± 2.9	24.9 ± 3	0.207	0.230	0.012
**Categories of MDS**	**All patients**	**Low sMg**	**High sMg**	***p* ^a^**	**r †**	***p* ^b^**
(a) Avoid foods	12 ± 2	12.3 ± 2.3	12 ± 2.6	0.506	−0.042	0.650
(b) Recommended foods	12 ± 3	11.3 ± 2.1	12.6 ± 3.2	**0.011**	0.319	**0.000**
(c) FVL	5 ± 2	4.5 ± 1.4	5.1 ± 2.3	0.122	0.231	**0.012**

Values are expressed as means ± SDs or medians (interquartile ranges). Significant differences or correlations are shown with bold numbers. HD, hemodialysis; PD, peritoneal dialysis; dMg, dialysate Mg concentration; LVMI, left ventricular mass index; ACEi, angiotensin-converting enzymes inhibitors; ARB, angiotensin II receptors blockers; CCB, calcium channel blockers; HDL, high-density lipoprotein; LDL, low-density lipoprotein; MIS, malnutrition–inflammation score; MDS, Mediterranean diet score; CRP, C-reactive protein; FVL, fruits, vegetables, legumes; † r, Pearson correlation coefficient between baseline characteristics and sMg. ^a^ the *p* value indicates the significance of the difference between the means of the two groups (low and high). ^b^ *p* value indicates the significance of the correlation (r) between sMg and the other variables in the study.

**Table 3:** Determinants of sMg.

Parameter	B	Standard Error	Standard Beta	*p*	Partial r
Constant	1.006	0.303		0.001	
dMg (0.25 vs. 0.50 mmol/L)	0.410	0.087	0.445	0.000	0.410
Recommended foods	0.032	0.011	0.241	0.005	0.262
HDL cholesterol	0.005	0.003	0.154	0.097	0.158
Serum albumin	0.063	0.062	0.086	0.308	0.097
ACEIs/ARBs	0.082	0.060	0.113	0.175	0.129
Dialysis mode (HD/PD)	0.251	0.078	0.346	0.002	0.293

dMg: Dialysate Mg concentration; HDL: High-density lipoproteins; ACEi: Angiotensin-converting enzymes inhibitors; ARB: Angiotensin II receptors blockers. Adjusted r^2^ = 0.29.

**Table 4:** Associations between baseline clinical and biochemical characteristics and MDS and Mediterranean diet food categories in our 117 dialysis patients.

		MDS	GROUP A ‘Avoid Foods’		GROUP B ‘Recommended Foods’		GROUP C ‘FVL’	
Factor	r	*p*	r	*p*	r	*p*	r	*p*
Diabetes	−0.073	0.432	0.050	0.593	−0.123	0.187	−0.200	**0.031**
LVMI	−0.206	**0.026**	−0.138	0.138	−0.105	0.261	−0.120	0.198
LVH	−0.218	**0.018**	−0.060	0.517	−0.195	**0.035**	−0.185	**0.045**
PVD	−0.324	**0.000**	−0.177	0.057	−0.237	**0.010**	−0.159	0.087
CAD	0.111	0.132	0.204	**0.027**	−0.059	0.525	−0.109	0.244
Stroke	−0.191	0.039	−0.077	0.389	−0.187	0.044	−0.273	0.003
SBP	−0.225	**0.015**	−0.159	0.088	−0.092	0.326	−0.081	0.324
DBP	−0.272	**0.003**	−0.063	0.500	−0.188	**0.045**	−0.160	0.085
HDL	0.224	**0.015**	0.136	0.144	0.076	0.417	0.161	0.084
LDL	−0.105	0.262	−0.062	0.507	−0.209	**0.019**	−0.091	0.230
sCa	−0.221	**0.017**	−0.136	0.144	0.396	0.000	−0.178	0.054
β-Blockers	−0.264	**0.004**	−0.023	0.805	−0.221	**0.017**	−0.201	**0.030**
Calcimimetics	0.190	**0.040**	−0.034	0.717	0.283	**0.002**	0.184	**0.047**

MDS: Mediterranean diet score; FVL: fruits, vegetables, legumes; LVMI: left ventricle mass index; LVH: left ventricle hypertrophy; PVD: peripheral vascular disease; CAD: coronary artery disease; SBP: systolic blood pressure; DBP: diastolic blood pressure; HDL: high-density lipoprotein; LDL: low-density lipoprotein; sCa: serum calcium. Bold indicates statistically significant values.

**Table 5:** Crude and adjusted hazard ratios of serum magnesium (per 1 mg/dL) for prediction of cardiovascular (CVD) and all-cause mortality in 117 patients with CKD stage 5D.

Model	Covariates	CVD Mortality HR (95% CI)	*p*	All-Cause Mortality HR (95% CI)	*p*
1	Crude risk of sMg (per 1 mg/dL)	0.17 (0.05–0.62)	0.007	0.27 (0.11–0.70)	0.007
2	1 + dMg	0.13 (0.03–0.51	0.004	0.23 (0.09–63)	0.004
3	2 + creatinine, albumin and ΜIS	0.10 (0.02–0.46)	0.004	0.19(0.07–0.54)	0.002
4	3 + age, PVD, diabetes and LVMI	0.12 (0.02–0.61)	0.011	0.26(0.09–0.74)	0.012
5	4 + MDS	0.20 (0.04–0.1.13)	0.069	0.27 (0.09–0.80)	0.018

dMg: dialysate Mg concentration; MDS: Mediterranean diet score; PVD: peripheral vascular disease; LVMI: left ventricle mass index; HR; hazard ratio.

**Table 6:** Crude and adjusted hazard ratios for prediction of cardiovascular (CVD) and all-cause mortality based on serum magnesium (sMg) levels above the median (High Mg) compared with levels below the median (Low Mg) in 117 patients with CKD stage 5D.

Model	Covariates	CVD Mortality HR (95% CI)	*p*	All-Cause Mortality HR (95% CI)	*p*
1	Crude risk of Low Mg vs. High Mg	0.34 (0.14–0.83)	0.018	0.51 (0.27–0.96)	0.037
2	1 + dMg	0.31 (0.13–0.76	0.011	0.47 (0.25–0.90)	0.023
3	2 + creatinine, albumin and ΜIS	0.32 (0.02–0.46)	0.020	0.42(0.22–0.83)	0.013
4	3 + age, PVD, diabetes and LVMI	0.34 (0.13–0.93)	0.036	0.43 (0.21–0.86)	0.018
5	4 + MDS	0.41 (0.15 –0.1.14)	0.088	0.44 (0.22–0.90)	0.024

dMg: dialysate Mg concentration; MDS: Mediterranean diet score.

**Table 7:** Crude and adjusted hazard ratios of serum magnesium (per 1 mg/dL) for prediction of cardiovascular (CVD) and all-cause mortality in 66 hemodialysis patients.

Model	Covariates	CVD Mortality HR (95% CI)	*p*	All-Cause Mortality HR (95% CI)	*p*
1	Crude risk of sMg (per 1 mg/dL)	0.12 (0.02–0.85)	0.034	0.19 (0.04–0.86)	0.031
2	1 + creatinine, sCa and albumin	0.07 (0.01–0.68)	0.022	0.14 (0.03–0.68)	0.015
3	2 + age, PVD and MIS	0.09 (0.01–0.92)	0.042	0.16 (0.03–0.98)	0.047
4	3 + Recommended food	0.17 (0.01–1.98)	0.157	0.25 (0.04–1.47)	0.125

sCa, serum Calcium; PVD, peripheral vascular disease; MIS, malnutrition–inflammation score; HR, hazard ratio.

**Table 8:** Crude and adjusted hazard ratios of serum magnesium (per 1 mg/dL) for prediction of cardiovascular (CVD) and all-cause mortality in 51 peritoneal dialysis.

Model	Covariates	CVD Mortality HR (95% CI)	*p*	All-Cause Mortality HR (95% CI)	*p*
1	Crude risk of sMg (per 1 mg/dL)	0.16 (0.03–0.98)	0.048	0.28 (0.08–0.96)	0.043
2	1 + Κ	0.09 (0.01–0.65)	0.017	0.21 (0.06–0.78)	0.019
3	2 + age, PVD, CAD and MIS	0.02 (0.02–0.62)	0.025	0.13 (0.02–0.72)	0.019
4	3 + Recommended food	0.07 (0.00–2.46)	0.144	0.18 (0.03–1.07)	0.160

K, serum potassium; PVD, peripheral vascular disease; CAD, coronary artery disease; MIS, malnutrition–inflammation score; HR, hazard ratio.

## Data Availability

The data supporting this study’s findings are available from the corresponding author upon reasonable request.
